# Plasmonic Gold Nanomaterials as Photoacoustic Signal Resonant Enhancers for Cysteine Detection

**DOI:** 10.3390/nano11081887

**Published:** 2021-07-23

**Authors:** Tsu-Wang Shen, Ting-Ku Ou, Bo-Yan Lin, Yi-Hsin Chien

**Affiliations:** 1 Department of Automatic Control Engineering, Feng Chia University, Taichung 40724, Taiwan; twshen@mail.fcu.edu.tw (T.-W.S.); pcabc7910051@gmail.com (T.-K.O.); 2 Master’s Program Biomedical Informatics and Biomedical Engineering, Feng Chia University, Taichung 40724, Taiwan; 3 Department of Materials Science and Engineering, Feng Chia University, Taichung 40724, Taiwan; xxx80450@gmail.com

**Keywords:** plasmonic Au nanomaterials, photoacoustic signals (PA signals), cysteine detection, PA resonant enhancers, portable photoacoustic system

## Abstract

The development of photoacoustic systems is important for the real-time detection of cysteine (Cys), a biothiol in biological systems that serves as a significant biomarker for human health. Advanced photoacoustic (PA) signals with colloidal plasmonic Au nanomaterials rely on the efficient conversion of light to energy waves under moderately pulsed laser irradiation. In this study, we synthesized Cys-capped Au nanorods (Au@Cys NRs) and Cys-capped Au nanoparticles (Au@Cys NPs) through a conjugate of three Cys concentrations (10, 100, and 1000 μM). These plasmonic Au nanomaterials can be used as a PA resonance reagent due to their maximum localized surface plasmon resonance (LSPR) absorption bands at 650 nm and 520 nm in Au NRs and Au NPs, respectively. Subsequently, the PA signals were noticeably increased proportionally to the concentrations in the Au@Cys NRs and Au@Cys NPs under 658 nm and 520 nm laser irradiation, respectively, according to our portable photoacoustic system. Furthermore, PA signal amplitudes in Cys detection are boosted by ~233.01% with Au@Cys NRs and ~102.84% with Au@Cys NPs enhancement, compared to free Cys, according to ultrasound transducers at frequencies of 3 MHz.

## 1. Introduction

Cysteine (Cys) is a crucial thiol-containing amino acid which plays a critical role in human pathologies [1,2], and is found in huge quantities in protein-rich foods [3]. In animal cells, Cys participates in many important and essential biological functions, including protein synthesis, detoxification, and metabolism [4]. Normally, the concentrations of Cys are maintained at around 30–200 μM for the synthesis of various proteins, serving as the source of sulfide in human metabolism [5]. A lack of Cys in the human body often leads to liver damage and edema, among other effects, while the presence of excessive Cys is associated with neurotoxicity, including Parkinson’s disease and Alzheimer’s disease [6]. In light of this, rapid and precise detection of Cys concentrations has become an extremely important issue in human pathologies [7,8]. In the past decade, a significant number of analytical methods have been reported for cysteine detection, including high-performance liquid chromatography (HPLC) [9,10], mass spectroscopy (MS) [11], electrochemical assay (or voltammetry) [12], spectrophotometry [13], and fluorescence spectrometry [8,14]. Nevertheless, these techniques remain somewhat restricted in terms of practical applications, as they are expensive and require skilled handling and operation. There is a strong need for a real-time, safe, affordable, easily applied detection method for Cys in vivo/vitro in many fields. The photoacoustic (PA) technique [15] is a potential candidate to provide noninvasive measurement of multiple substances in vivo/vitro [16] with high resolution and contrast [16,17], deeper penetration, and easy detection, which has been widely used in various biomedical sensing, bio-imaging [15,18], and biomedicine applications [19] by converting the absorbed photon energy to ultrasonic emissions.

The PA technique was developed quite rapidly by several early adopters of photoacoustic imaging because of its noninvasive, high-resolution, and unique photo-to-acoustic spectrum characteristics. This technology effectively relies on the generation and detection of ultrasound (PA waves) via thermoelastic expansion of targets that absorb laser pulses [8]; that is, it measures the conversion of electromagnetic energy into acoustic pressure waves. Fundamentally, the analyte is irradiated with a pulsed laser, resulting in the generation of an ultrasound wave due to optical absorption and rapid thermal expansion of an analyte [20]. The initial pressure, p0, generated by an optical absorber, is described in Equation (1):

p0 = Tμ_a_F (1)

where F is the laser fluence at the absorber, μ_a_ is the optical absorption coefficient, and T is the Grüneisen parameter of the analyte [21]. The light is absorbed and converted to an outgoing PA wave that can be detected by an ultrasound transducer, and collected as PA signals. A new concept that combines ultrasound and resonant materials to positively resonate PA waves under appropriate wavelength light irradiation is an important recent finding [22]. The resonant materials for PA signal enhancement are based on optical-to-acoustic conversion efficiency by the incorporation of various resonating agents. There are several resonating agents, such as fluorescent dyes [23,24], metals, and plasmonic nanoparticles [25,26], that have been introduced for the better PA signal enhancement of analytes. In particular, plasmonic metal nanoparticles, such as gold (Au) and silver (Ag) nanoparticles (NPs), have been suggested as resonant nanomaterials due to their unique surface plasmon resonance (SPR) optical properties. In metallic nanoparticles, the collective oscillation of the free electron-induced local temperature and resonating energy field increase on the surrounding surface for potential applications in photoelectronics, surface-enhanced Raman scattering (SERS), photoacoustic imaging, and SPR-based sensing. In general, Au NPs have high biocompatibility, great structural stability, reproducible synthesis methods, and a high-value extinction coefficient in the visible region. The tunable SPR absorption band is based on particle sizes and shapes, allowing them to be highly efficient energy absorbers at a suitable wavelength of light, in order to generate the local thermal effect and energy resonance field. For instance, Kim et al. demonstrated optical absorbing gold nanocages (Au NCs) with [Nle4, D-Phe7]-α-melanocyte-stimulating hormone conjugation. The bioconjugated Au NCs, as a contrast agent, have shown extremely high-resolution photoacoustic tomography (PAT) for in vivo melanoma bioimaging applications [27]. Yang et al. also reported poly(ethylene glycol)-coated Au NCs as a potential near-infrared (NIR) contrast agent to enhance the optical absorption in the in vivo photoacoustic tomography of a rat cerebral cortex [28]. Au nanorods (Au NRs), Au NCs, and nanoshells with high tunable optical absorption cross-sections have been successfully demonstrated in photoacoustic imaging (PAI) and PA signal applications [25,29,30].

In this study, we present the investigation and design of a photoacoustic system for cysteine molecule concentration detection based on PA signals, with SPR-related Au nanomaterials. The plasmonic Au nanomaterials showed highly optical absorption cross sections, to enlarge the heat and energy transfer from the Au nanomaterials to the outer Cys molecule with appropriate light irradiation. Therefore, we synthesized plasmonic Au NRs (aspect ratio ~2.4) and Au NPs (13 nm), with a maximum SPR absorption band at 650 nm of Au NRs and 520 nm of Au NPs. Both Au nanomaterials were conjugated with different concentrations of Cys (10, 100, 1000 μM) to form Au@Cys NPs and Au@Cys NRs, which have been used as photoacoustic resonant enhancers and can evaluate the PA signals of Cys molecule species under 520 nm and 658 nm laser irradiation, respectively. Hence, the aim of this study was to demonstrate that plasmonic Au nanomaterials may potentially be a PA signal enhancer for Cys detection, and that PA signal features can be used to calculate Cys concentrations, which can potentially be applied in real-time detection and identification of analytes.

## 2. Materials and Methods

### 2.1. Materials and Instruments

The absorption spectra were acquired on Edinburgh Instruments FS5 (Edinburgh Instruments Ltd., Livingston, UK). The FTIR and XPS analysis were obtained with an FTIR SPECTROMETER FRONTIER (Perkin Elmer Inc., Waltham, MA, USA) and PHI Hybrid Quantera (ULVAC-PHI). The concentration of Au nanomaterials was measured with an OPTIMA 2000DV (Perkin Elmer Inc., Waltham, MA, USA), and TEM images were obtained using a JEM-1400 (JEOL Ltd., Akishima, Tokyo, Japan). Hydrogen tetrachloroaurate (III) trihydrate (HAuCl_4_·3H_2_O, ≥99.9%) and cetyltrimethylammonium bromide (CTAB, ≥99%) were purchased from Alfa Aesar (Heysham, Lancashire, UK). Sodium citrate dihydrate (≥99%) was acquired from Avantor Inc. (Radnor, PA, USA). Sodium borohydride (NaBH_4_, ≥99%) was obtained from Koch-Light Laboratories Ltd. (Haverhill, England, UK). Sulfuric acid (≥98%) and L-ascorbic acid (≥99%) were purchased from Fluka (Charlotte, NC, USA). Silver nitrate (AgNO_3_, 99.85%) was obtained from Acros Organics (Waltham, MA, USA). L-cysteine (≥97%) was purchased from Merck KGaA Ltd. (Darmstadt, Germany). Ultrapure deionized water (18.3 MΩ·cm^−1^, RODA, Te Chen Instruments CO., Ltd., Taichung, Taiwan) was used for all solution preparations. All chemicals were used as received, without further purification.

### 2.2. Synthesis of Gold Nanospheres (Au NPs)

A total of 13 nm of Au NPs was used, following the literature with slight modifications [31]. An Au solution (0.56 mM) in a three-neck flask was prepared by adding 5 mL HAuCl_4_·3H_2_O (5 mM) to 40 mL DI-water. Subsequently, the stock solution was heated to 100 °C, with vigorous stirring, until boiling. Then, 5 mL tri-sodium citrate (38.8 mM) solution was quickly injected into the boiling Au solution with stir bar stirring at 100 °C for another 10 min. During the reaction time, the color of the solution turned from colorless to dark purple to purple-red. After 10 min, the solution was cooled to room temperature, and the Au NPs were collected by centrifugation at 10,000 rpm for 10 min.

### 2.3. Synthesis of Gold Nanorods (Au NRs)

(i) Synthesis of Gold Seeds (Au Seeds)

The Au NRs were synthesized following the literature, with slight modifications [32]. There are two steps in Au NR synthesis: Au seed formation and Au growth solution preparation. First, the Au seeds were prepared by mixing 100 μL HAuCl_4_·3H_2_O (24 mM) and 7.5 mL CTAB (0.1 M) with vigorous stirring. Then, a fresh preparation of NaBH4 (600 μL, 10 mM) solution was quickly injected into the above-described solution. The solution color immediately changed from light yellow to brown, and the solution was stirred for another 30 min at room temperature.

(ii) Synthesis of Gold Growth Solution and Au NRs

The Au growth solution was prepared by CTAB (10 mL, 0.1 M) and HAuCl_4_·3H_2_O (0.204 μL, 24 mM) in a sample vial. Next, a solution of 0.2 mL H_2_SO_4_ (0.5 M), 50 μL AgNO_3_ (10 mM) and 80 μL ascorbic acid (0.1 M) was added to the sample vial, with stir bar stirring. After a few minutes, 24 μL of the Au seed solution from step (i) was quickly added to the Au growth solution with vigorous stirring to form Au NRs for another 12 h (or overnight) at room temperature. Finally, the Au NRs were collected by 7200 rpm centrifugation for 10 min.

### 2.4. L-Cysteine Capped Au Nanomaterials (Au@Cys NRs and Au@Cys NPs)

Three concentrations (10, 100, 100 μM) of Cys were prepared to conjugate with Au NPs and Au NRs. Each Eppendorf tube contained 0.5 mL Au NPs (or Au NRs) with 200 ppm, and each one was added to a solution of 0.5 mL L-cysteine (0, 20, and 200 μM) separately. Then, all Eppendorf tubes were placed onto the vortex with vigorous mixing for 2 h at room temperature for further Cys detection in a photoacoustic system.

### 2.5. Photoacoustic System

**Scheme 1:**
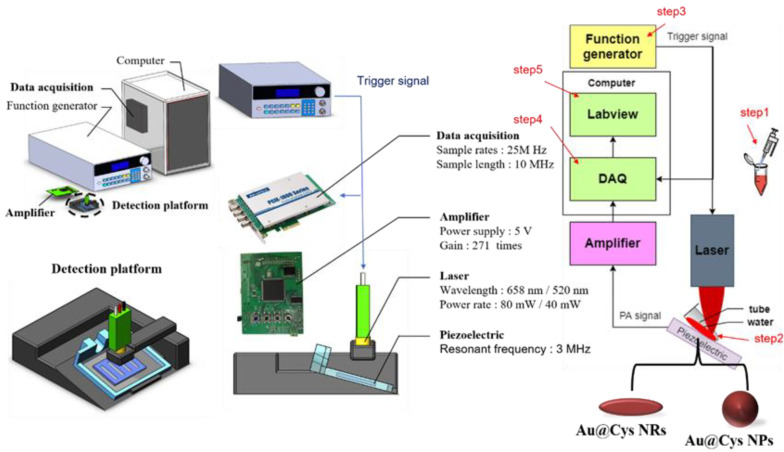
Photoacoustic system, comprising a custom-made piezoelectric, an amplifier, and continuous class III 520 nm/658 nm lasers.

The photoacoustic system consisted of a 3 MHz centered frequency piezoelectric (STEMINC Inc., Davenport, FL, USA) coating with polydimethylsiloxane (PDMS), an amplifier with a gain of 271 times, and class IIIB 520 nm/658 nm lasers controlled by transistor–transistor logic (TTL) signals, as shown in Scheme 1 and Figure S1 (photograph of a portable PA system). The received echo ultrasound signals were digitized by a data acquisition card (PCIE-1840 card, Advantech^TM^ Inc., Taipei, Taiwan) with a 25 MHz sampling rate, cable-linked to a computer to avoid aliasing; the data were recorded by LabView^TM^ (R2019, National Instruments Inc., Austin, TX, USA). The photoacoustic signals were analyzed by MATLAB^TM^ (R2020 version MathWorks Inc., Natick, MA, USA). The actual operation of the system used laser excitation pulses at a 50% duty cycle over an 8 ms period on samples in tubes (Sorenson BioScience Inc., 0.2 mL PCR Tubes With Attached Caps, Salt Lake City, UT, USA), and then ultrasonic resonant signals were recorded for analysis. Instead of placing the tube in water, the tube was placed directly on a water-moistened ultrasound transducer. The experiment comprised five steps: (1) place a sample into the cover slip; (2) place the sample object on a 3D-printed platform and set the laser on the top position, 6 mm away from the tube, by focusing it as a small spot of 1.5 mm diameter after irradiation; (3) control the TTL signals to turn the laser on or off; (4) capture the photoacoustic signals using a data acquisition card; (5) process “on” signals to generate their histograms for analysis.

Signal processing is essential to enhance the signal-to-noise ratio of PA signals. The PA signals were sampled at 25 MHz and contained a large amount of information. Due to memory limitations, 50 PA signals triggered by TTL signals were recorded on the data acquisition card. We repeated this step four times to collect the 200 PA signals. We further filtered out 60 Hz interference and eliminated 2% outliner PA signals to ensure the signal quality. Each PA signal was then segmented into on/off sessions. Two features were extracted from the on-session PA signals: the amplitudes and histogram density distribution widths of the PA signals. The histogram method, first introduced by Pearson [33], divides the entire range of on-session values into a series of intervals (0.01 mV) and then counts how many values fall into each interval. Histograms give a rough sense of the density of the underlying data distribution. The histograms of PA signals also provide mean amplitude (M) and histogram density distribution width (l) features, which can be used for further analysis. The threshold of the density distribution width was set at 40 according to our previous results [34]. Hence, the analysis data were based on the features from 196 PA signals, and the signal average method [35] superposed those PA signals to form our example signals.

## 3. Results and Discussion

**Figure 1:**
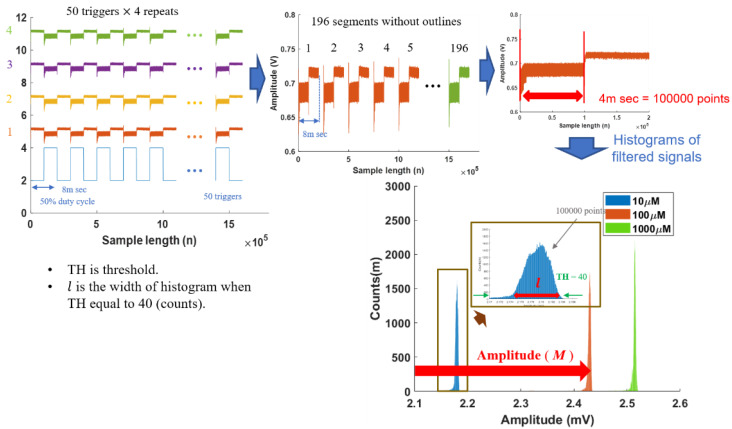
Photoacoustic (PA) signal processing steps to generate histograms.

**Figure 2:**
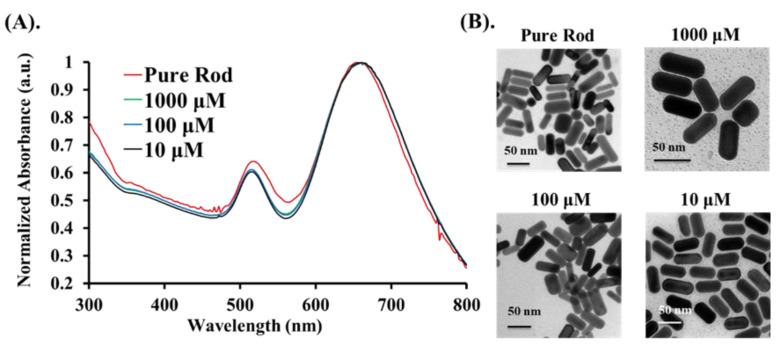
Different concentrations (10 μM, 100 μM, and 1000 μM) of Au@Cys NRs: (**A**) UV–VIS absorption spectrum, and (**B**) TEM images.

The experimental analysis procedure for measuring the PA signals from the Cys is shown in Figure 1. Statistical analyses were carried out with Prism GraphPad (version 5, GraphPad Software Inc., San Diego, CA, USA) and MATLAB software. PA features were analyzed among control parameters using a one-way ANOVA with Bonferroni’s multiple comparison post-hoc test to provide the interactive relationships between the factors and the three concentrations. Three sets of parameters were controlled in this study, including two laser excitation wavelengths (658 nm and 520 nm), three substances (Cys, Au@Cys NRs, and Au@Cys NPs), and three Cys concentrations (10, 100, and 1000 μM). The relationships between PA signal features and the three sets of parameters were the focus of this study. First, we chose the Au NRs with an average aspect ratio of 2.4 as a PA signal enhancer for Cys sensing. As shown in Figure 2, Au NRs dispersed in H_2_O with an average length and width of ∼47.5 nm and ∼19.4 nm, respectively, were measured by using transmission electron microscopy (TEM) and showed a typical longitudinal SPR maxima band at 650 nm. Three different Cys concentrations were conjugated onto the surface of Au NRs to form the Au@Cys NRs by thiol–Au bonding (Au-S bond). TEM images (Figure 2B) of Au@Cys NRs showed no change in morphology, and the longitudinal SPR band remained at 650 nm (Figure 2A). Meanwhile, Fourier-transform infrared spectroscopy (FTIR) analysis provided information on the surface moiety and additional evidence of the conjugation of Au NRs with Cys (Figure S2) [36].

**Figure 3:**
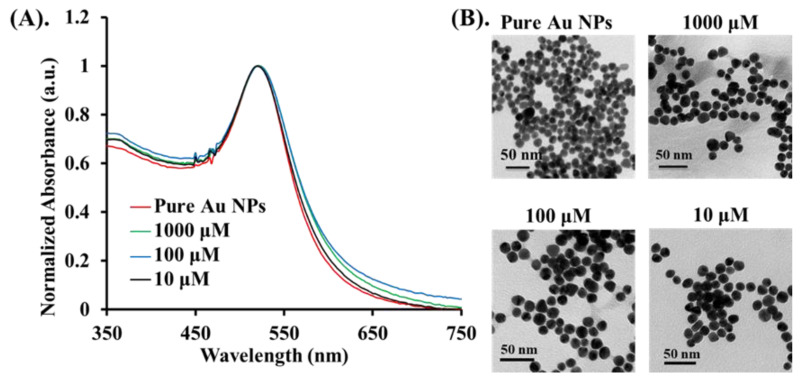
Different concentrations (10 μM, 100 μM, and 1000 μM) of Au@Cys NPs: (**A**) the UV–VIS absorption spectrum, and (**B**) TEM images.

The main objective of this study was to evaluate the PA signal enhancement on Cys molecules provided by targeting Au NPs and Au NRs. Since the PA amplitude is proportional to the optical absorption, a strong increase in the PA signal was expected at the SPR band of the Au nanoparticles, depending on size, shape, and environment. Therefore, we synthesized two types of Au nanomaterials (Au NPs and Au NRs) with different sizes and shapes [37]. The Au NPs with an average size of 13 nm were prepared by using the thermal method, and showed an SPR maxima band of 520 nm by UV–VIS spectrometer measurement. Next, three concentrations of Cys were conjugated to the surface of Au NPs to form the Au@Cys NPs. As seen in Figure 3, there was no morphology change in the TEM images of the Au@Cys NPs, and the SPR band remained at 520 nm [38,39]. The FTIR analysis of Au@Cys NPs is shown in Figure S3 [36,40]. Due to the presence of Cys conjugation on the surface of the Au NRs and Au NPs, we took more X-ray photoelectron spectroscopy (XPS) measurements of the Au 4f core-level binding energy, which is sensitive to the chemical environment. For the XPS analysis of Au@Cys NPs, the Au region displayed two splitting peaks at 81.35 eV and 85.05 eV, corresponding to Au 4f_7/2_ and 4f_5/2_ (Figure S4), respectively. The peaks shifted 0.2 eV to a higher binding energy, relative to single crystalline Au NPs [41,42]. The upward chemical shift indicated that the electrons transferred from the protonation amine (NH_3_^+^) of the Cys to the Au surface. Thus, the Cys molecule conjugation onto the Au surface was proven by a combination of the results of the FTIR and XPS analysis.

**Figure 4:**
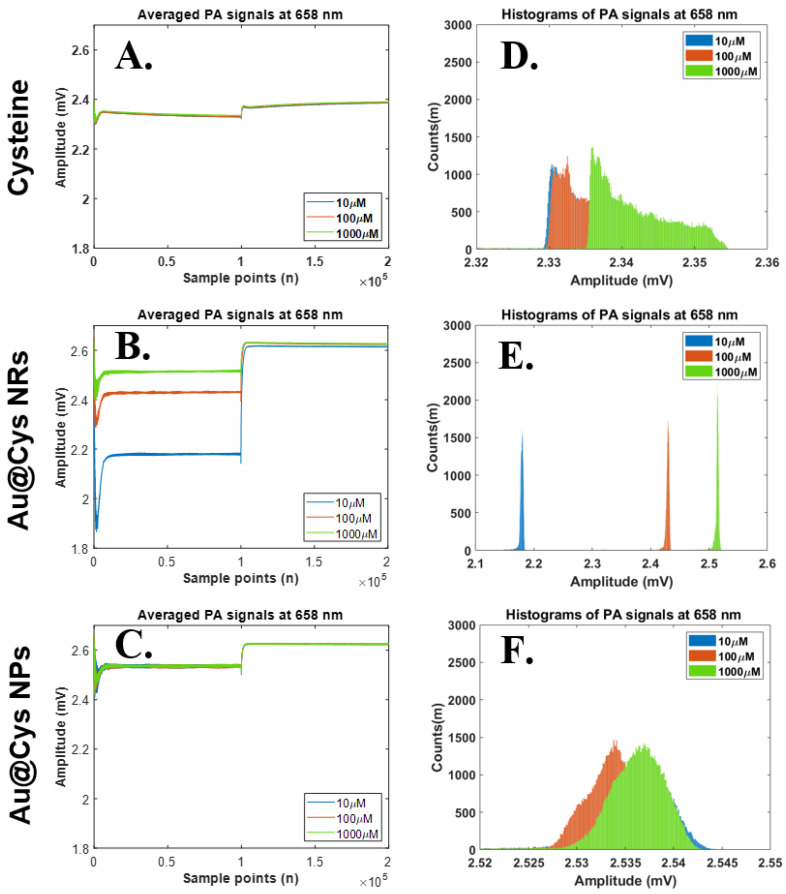
Relationships of time domain PA signals (upper) and their histograms (lower) at the laser wavelength 658 nm. Statistical analysis results of histogram widths among three parameter sets, including 658 nm laser excitation, three substances (Cys, Au@Cys NRs and Au@Cys NPs), and three Cys concentrations (10, 100, and 1000 μM). (**A**–**C**) Time domain PA signals from Cys, Au@Cys NRs, and Au@Cys NPs samples, respectively; (**D**–**F**) Histogram plots of PA signals (**A**–**C**) from Cys, Au@Cys NRs, and Au@Cys NPs samples, respectively.

The PA signals of three samples (Cys, Au@Cys NRs, and Au@Cys NPs), containing different concentrations of Cys (10, 100 and 1000 μM), were able to be collected by the photoacoustic system under a 658 nm (80 mW) diode laser for 4 ms pulse width. The detailed results of PA signals and their histograms generated are shown in Figure 4. The results represent time domain PA signals from Cys, Au@Cys NR, and Au@Cys NP samples, respectively (Figure 4A–C). The histograms of the PA signals from Cys, Au@Cys NR, and Au@Cys NP samples are shown in Figure 4D–F, respectively. The results show that the changes of the PA amplitudes and histogram widths of Cys and the Au@Cys NPs were subtle for the three different concentrations. The PA amplitudes and histogram widths obviously increased proportionally to the concentrations in the Au@Cys NRs, but not in the Cys samples and Au@Cys NPs. Compared with the Cys samples and Au@Cys NPs, the histograms of the Au@Cys NRs samples were separated effectively. That is, the histograms of Cys concentrations can be significant identified by Au@Cys NRs under 680 nm laser treatment. However, this did not occur for the Au@Cys NPs samples under 658 nm diode laser irradiation. The Cys PA signals were enhanced due to the plasmonic optical properties in Au NRs, as described in Figure 3A; the localized surface plasmon resonance (LSPR) of the Au NRs, with the transverse and longitudinal mode, were located at 520 nm and 650 nm. Therefore, the PA signals of Au@Cys NRs were significantly improved compared to Cys and Au@Cys NPs due to the longitudinal absorbing mode of Au NRs at 650 nm, which matched the excitation wavelength at 658 nm in this experiment [32]. When the Au NRs were illuminated with a 658 nm laser, the electromagnetic energy was highly localized near the metal−dielectric interface and was therefore strongly coupled with the vibration mode of Cys on the Au NRs’ surfaces [43]. The 3 MHz-centered ultrasound transducer received stronger resonance from Au@Cys NRs compared with Au@Cys NPs and Cys. Consequently, the PA signals of the Au@Cys NRs, enhanced by various Cys concentrations, increased. However, the PA amplitudes and histogram widths did not show much difference in Cys and Au@Cys NPs under the 658 nm laser irradiation due to the off-resonance excitation wavelength. The morphology of the Au@Cys NRs and Au@Cys NPs, and their SPR behavior, were observed to show no change after laser treatment (Figure S5).

**Figure 5:**
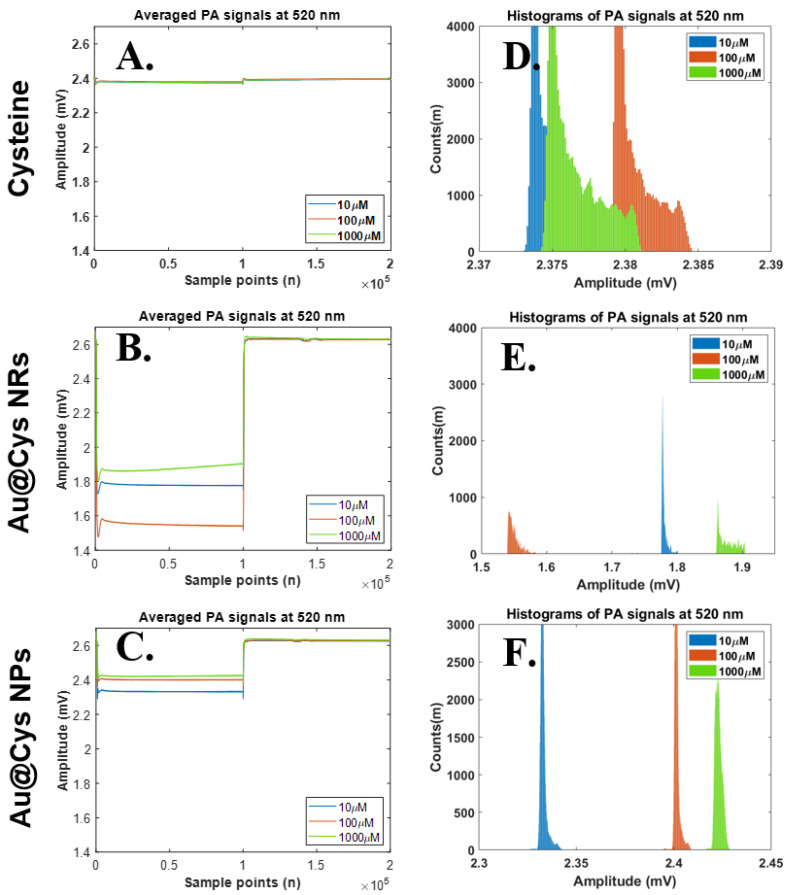
Relationships on time domain PA signals (upper) and their histograms (lower) at laser wavelength 520 nm. Statistical analysis results of histogram widths among three parameter sets, including 520 nm laser excitation, three substances (Cys, Au@Cys NRs and Au@Cys NPs), and three Cys concentrations (10, 100, and 1000 μM). (**A**–**C**) Time domain PA signals from Cys, Au@Cys NRs, and Au@Cys NPs samples, respectively; (**D**–**F**) Histogram plots of PA signals (**A**–**C**) from Cys, Au@Cys NRs, and Au@Cys NPs samples, respectively.

Similarly, we further changed the laser wavelength from 658 nm to 520 nm for Cys detection in the same samples. The PA signals and their histograms for Cys, Au@Cys NPs, and Cys@Au NRs were collected under 520 nm laser irradiation (40 mW) for 4 ms per pulse. The results for the PA amplitudes and histogram widths are shown in Figure 5. As can be seen in Figure 5A,D, the PA amplitudes and histogram widths did not show any change for the Cys samples. The PA signals clearly changed under 520 nm laser treatment for Au@Cys NRs due to the transverse absorbing mode of Au NRs being 520 nm, but this was not proportional to the Cys concentrations; it can be speculated that a number of Cys molecules randomly conjugated onto a transverse site of Au NRs. (Figure 5B,E). By comparison, the Au@Cys NPs separated the histograms proportionally to different concentrations, as can be seen in Figure 5C,F. An increase in the PA wave and histogram of Au@Cys NPs was also attributed to the matching resonance band of Au NPs at 520 nm; the surface electrons oscillated together with a regular orientation to enhance the local energy, inducing the PA wave of Cys molecules by using a 3 MHz-centered ultrasound transducer [43,44]. In short, the Cys samples with different Cys concentrations showed the similar amplitudes under 520 nm and 658 nm laser irradiation. For comparison, the samples of Au@Cys NPs and Au@Cys NRs demonstrated significantly difference amplitudes corresponding to Cys concentrations with 520 nm and 658 nm laser treatment, respectively.

One-way ANOVA was applied to statistic multiple laser-pulse measurements (N = 196) among three concentrations (10 μM, 100 μM, and 1000 μM) by using three types of samples (Cys, Au@Cys NRs, and Au@Cys NPs) and two wavelengths to determine the means and their standard deviation. Bonferroni’s multiple comparison test was applied as a post-hoc test to provide the interactive relationships between the factors and three concentrations. Table 1 shows the analysis results of the amplitude (M) and histogram widths (l) with different concentrations. Without Au nanomaterials as an enhancer, the Cys can somewhat be excited by a 658 nm laser, with a significant difference only for concentrations of 10 μM and 1000 μM. The Au@Cys NRs excited by a 658 nm laser showed significant propositional differences at all three concentrations. This indicated that Au@Cys NRs increased the PA signal resolution compared with Cys only (also in Figure 4E). Moreover, the Au@Cys NPs excited by a 520 nm laser showed significant propositional differences among all three concentrations. That is, the plasmonic Au nanomaterials were able to act as a resonant agent to obtain high-resolution PA signals of Cys through proper wavelength laser treatment, compared to PA signals of Cys without Au nanomaterials. Compared with Cys, at a 658 nm laser irradiation, the absolute values of the PA features increased by 6.01% (average amplitudes of Au@Cys NRs-Cys/Cys from the three concentrations), and 233.01% (average on the histogram of Au@Cys NRs-Cys/Cys from the three concentrations) in the average amplitude and histogram width after Au NRs addition; at a 520 nm laser, the absolute values of the PA features increased by 1.54% and 102.84% in average amplitude and histogram width after Au NPs addition, respectively. Table 1 shows the results that demonstrate that the PA signal features were significantly proportional to the Cys concentrations if the SPR mode of Au nanomaterials matched their specific wavelengths.

**Figure 6:**
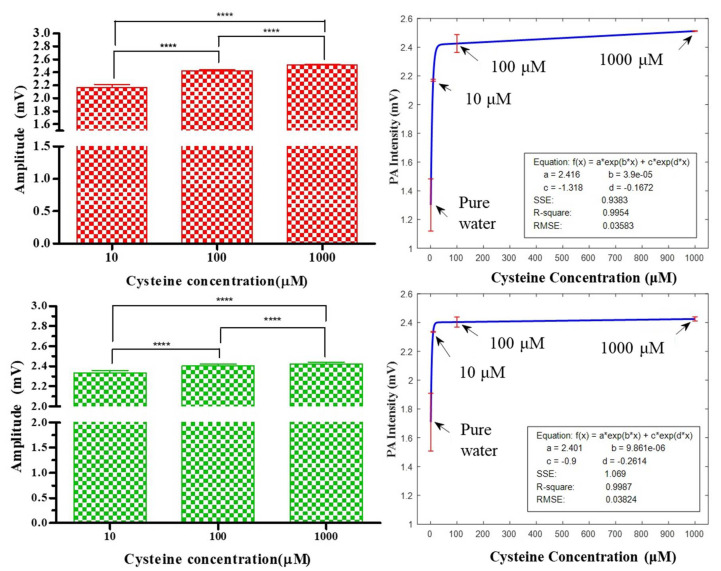
The red and green color bars represent the PA amplitudes of Au@Cys NRs and Au@Cys NPs irradiated by 658 nm and 520 nm lasers, respectively. The fitted curves summarize the relationships between the amplitude intensities and four concentrations (pure water, 10, 100, and 1000 μM). The means and standard deviations are plotted according to a series of PA amplitudes (N = 196) for the best fit. **** Significantly *p* < 0.0001.

According to the one-way ANOVA, the results showed that the amplitudes of the PA signals were significantly proportional to the Cys concentrations when the SPR mode of the Au nanomaterials matched the specific wavelengths in Figure 6. Hence, plasmonic Au nanomaterials could be a potential PA resonant enhancer for Cys detection. However, the amplitude differences among 10, 100, and 1000 μM is nonlinear, and the amplitude differences reduce when concentrations increase. Therefore, formulas exist that represent the relationships between the signal intensities and concentrations. Fitted curves can be used to summarize these relationships. The relationship curves are provided in Figure 6, demonstrating the effective measurement of the cysteine concentration based on PA amplitude intensities. The PA amplitude of pure water was set as the calibration factor among different experiment settings for signal background reference. A series of PA amplitudes (N = 196) provided mean and standard deviation plots for four Cys concentrations (pure water, 10, 100, and 1000 μM) and had the best fit to the exponential formulas in Figure 6. For a 658 nm laser treatment applied to Au@Cys NRs, the curve fitting formula had an R-square value of 0.9945, with a mean square error (MSE) of 0.03583. In addition, for a 520 nm laser irradiation applied to Au@Cys NPs, the curve fitting formula had an R-square value of 0.9987, with an MSE of 0.03824. In regression, the R-square coefficients of determination indicate the goodness of fit of the prediction model to approximate the real data points. Our R-square values showed that the regression predictions were nearly perfect to fit the data [45].

## 4. Conclusions

In summary, we proposed the use of plasmonic Au nanomaterials to amplify the PA signals of cysteine by applying both the amplitude and histogram method analysis to our portable noninvasive photoacoustic system. Cys-capped Au NR and Au NP colloids in water can act as a PA wave resonant enhancer under suitable LSPR wavelengths, with regard to Au NRs and Au NPs at 658 nm and 520 nm laser treatment. Proportional PA signal enhancement with certain Cys concentrations (10, 100, and 1000 μM) could be applied to conjugate plasmonic Au nanomaterials through efficient conversions of light to energy fields, due to electron resonance across the surface of Au nanomaterials. By comparison, the absolute values of the PA features of Cys at the three concentrations increased by 6.01% at the 658 nm wavelength and increased by 1.54% at the 520 nm wavelength. Surprisingly, the absolute values of Cys PA features dramatically increased by 233.01% (average on the histogram of Au@Cys NRs-Cys/Cys from the three concentrations) with Au@Cys NRs at 658 nm laser irradiation, and by 102.84% with Au@Cys NPs at 520 nm. Hence, we demonstrated a suitable and highly stable PA signal enhancer for precise Cys concentration detection with our potentially portable PA system, which may be applied to real-time Cys-biosensors in specimens.

## Figures and Tables

**Table 1:** Means and standard deviations of PA features (including amplitude (M) and histogram width (l)) with Bonferroni’s multiple comparison post-hoc test, to provide the interactive relationships between the factors and three concentrations.

Amplitude (M) N = 196							
Mean ± SD (mV) ^(1)^					*p* Value		
CONC (μM)	Cys	Cys@Au NRs	Cys@Au NPs	CONC vs. CONC	Cys	Cys@Au NRs	Cys@Au NPs
658 nm laser				658 nm laser			
10	2.3367 ± 0.0005	2.1691 ± 0.0070	2.5338 ± 0.0015	10 vs. 100	ns	****	****
100	2.3367 ± 0.0066	2.4251 ± 0.0621	2.5324 ± 0.0020	10 vs. 1000	****	****	****
1000	2.3416 ± 0.0107	2.5117 ± 0.0014	2.5346 ± 0.0032	100 vs. 1000	****	****	****
520 nm laser				520 nm laser			
10	2.3757 ± 0.0038	1.7860 ± 0.1144	2.3350 ± 0.0019	10 vs. 100	****	****	****
100	2.3807 ± 0.0108	1.5584 ± 0.07012	2.4030 ± 0.0351	10 vs. 1000	*	***	****
1000	2.3767 ± 0.0070	1.8821 ± 0.3577	2.4244 ± 0.0145	100 vs. 1000	****	****	ns
**Histogram Width (l) N = 196**							
**Mean ± SD (mV) ^(1)^**					***p* Value**		
**CONC** **(μM)**	**Cys**	**Cys@Au NRs**	**Cys@Au NPs**	**CONC** **vs. CONC**	**Cys**	**Cys@Au NRs**	**Cys@Au** **NPs**
658 nm laser				658 nm laser			
10	9.3034 ± 0.4402	31.9986 ± 5.0176	46.7316 ± 3.3908	10 vs. 100	**	****	ns
100	9.4693 ± 0.6127	33.6338 ± 4.8570	46.7199 ± 3.3764	10 vs. 1000	****	****	****
1000	12.0049 ± 0.9178	35.9976 ± 4.9866	53.4999 ± 4.1349	100 vs. 1000	****	****	****
520 nm laser				520 nm laser			
10	7.6219 ± 0.7148	14.3424 ± 6.1438	15.3343 ± 6.0414	10 vs. 100	ns	****	ns
100	7.6477 ± 0.7394	15.6709 ± 6.0875	15.3304 ± 6.0847	10 vs. 1000	****	****	****
1000	8.3673 ± 0.5976	18.0120 ± 6.5396	17.3117 ± 6.0219	100 vs. 1000	****	****	****

* Significantly *p* < 0.05, ** Significantly *p* < 0.01, *** Significantly *p* < 0.001, **** Significantly *p* < 0.0001; ^(1)^ The number of “Mean ± SD (mV)” were shown into the fourth significant digits after the decimal point.

## Data Availability

Data is contained within the article or supplementary material.

## Supplementary Materials

Supplementary data associated with this article, including FT-IR analysis, XPS analysis, and proposed system for Cys measurement, can be found in the online version. The following are available online at https://www.mdpi.com/article/10.3390/nano11081887/s1. Figure S1. The proposed system for Cys measurement. (A). The self-designed FPGA card to drive Class III laser diode and to amplify PA signals; (B). The tube placed on top of the ultrasound transducer to correct PA signals; Figure S2. FT-IR spectra includes pure *L*-cysteine (green line) and as-prepared Au NRs (red line) and Au@Cys NRs (blue line). The IR band at 2850 and 2915 cm^−1^ are assigned to C-H stretching and around 3176 cm^−1^ and 3440 cm^−1^ are associated with asymmetric stretching of OH groups for Au NRs. For *L*-cysteine, the characteristic FT-IR peak of absorption peaks at 1392 cm^−1^ and 1577 cm^−1^ are assigned to asymmetric and symmetric stretching of COO^-^ respectively. The peak at 1535 cm^−1^ and 2549 cm^−1^ are attributed to N-H stretching and S-H stretching. The spectrum of Au@Cys NRs exhibited the N-H band is shifted to 1586 cm^−1^ because *L*-cysteine is modified on Au NRs and the S-H band is dispersed which converted to a strongly Au-S bond; Figure S3. FT-IR spectra of pure *L*-cysteine (green line), as-prepared Au NPs (red line) and Au@Cys NPs. The peaks at 1388 cm^−1^ and 1561 cm^−1^ are assigned to asymmetric and symmetric stretching of COO^-^ and is slightly shifted in the Au@Cys NPs condition. Then, the vibration curve of N-H band in Au@Cys NPs is shifted to 1578 cm^−1^ because of the *L*-cysteine conjugation onto Au NRs. Also, the S-H bond in the *L*-cysteine is dispersed which converted to a strongly Au-S bond; Figure S4. The XPS spectrum of Au 4f core-level of (A) Au NRs and (B) Au@Cys NRs; Figure S5. The UV-Vis absorbance spectrum and TEM images (insert) with four Cys concentrations (0 μM (pure Au), 10 μM, 100 μM and 1000 μM) of (A) Au@Cys NPs and (B) Au@Cys NRs after 520 nm and 650 nm laser irradiation within 4 ms.
