# Supplementary material for: Plasmonic Gold Nanomaterials as Photoacoustic Signal Resonant Enhancers for Cysteine Detection
Source: Nanomaterials (Basel). 2021 Jul 23;11(8):1887. doi: 10.3390/nano11081887 (PMC8401226; doi:10.3390/nano11081887)
Supplement: Supplementary file 1 [file nanomaterials-11-01887-s001.zip › nanomaterials-1278278-supplementary.pdf]

nanomaterials

Supplemental Information

Plasmonic Gold Nanomaterials as Photoacoustic Signal Resonant Enhancers for Cysteine Detection

Tsu-Wang Shen [1,2], Ting-Ku Ou [1], Bo-Yan Lin [3] and Yi-Hsin Chien [3,*]

1 Department of Automatic Control Engineering, Feng Chia University, Taichung, 40724, Taiwan; twshen@mail.fcu.edu.tw (T.-W.S.); pcabc7910051@gmail.com (T.-K.O.)
2 Master's Program Biomedical Informatics and Biomedical Engineering, Feng Chia University, Taichung, 40724, Taiwan
3 Department of Materials Science and Engineering, Feng Chia University, Taichung, 40724, Taiwan; xxx80450@gmail.com (B.-Y.L.)
* Correspondence: yhchien@fcu.edu.tw; Tel.: +886-424517250 Ext. 5316

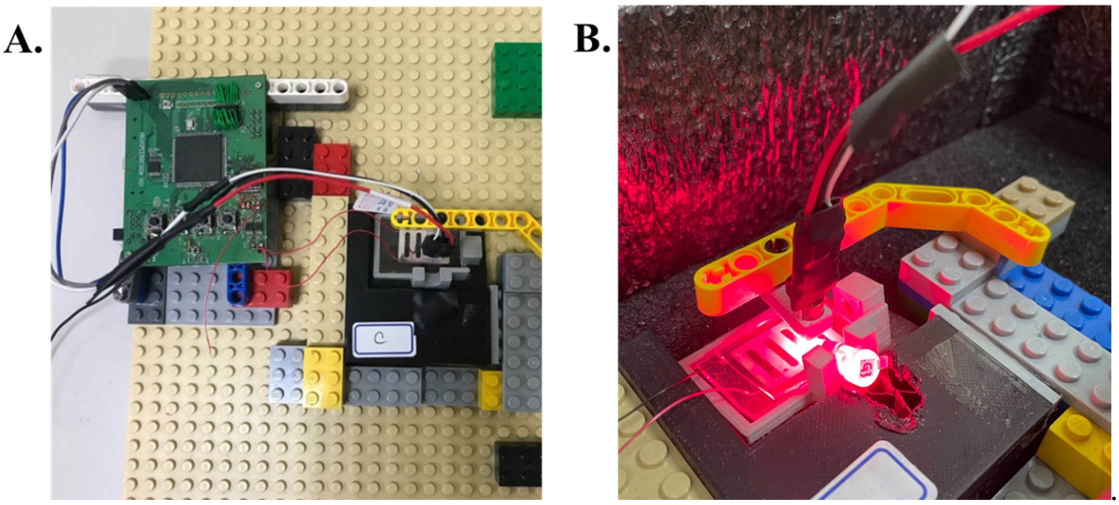

Figure S1. The proposed system for Cys measurement. **(A)**. The self-designed FPGA card to drive Class III laser diode and to amplify PA signals; **(B).** The tube placed on top of the ultrasound transducer to correct PA signals.

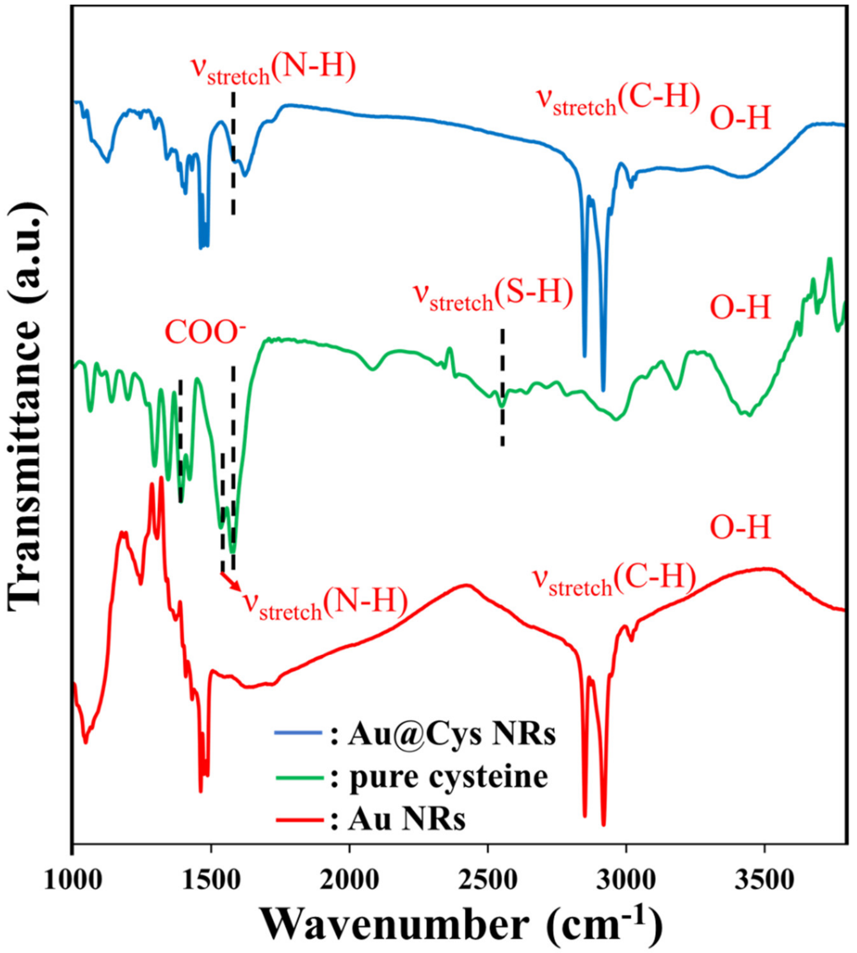

Figure S2. FT-IR spectra includes pure *L*-cysteine (green line) and as-prepared Au NRs (red line) and Au@Cys NRs (blue line). The IR band at 2850 and 2915 cm^{-1} are assigned to C-H stretching and around 3176 cm^{-1} and 3440 cm^{-1} are associated with asymmetric stretching of OH groups for Au NRs. For *L*-cysteine, the characteristic FT-IR peak of absorption peaks at 1392 cm^{-1} and 1577 cm^{-1} are assigned to asymmetric and symmetric stretching of COO^- respectively. The peak at 1535 cm^{-1} and 2549 cm^{-1} are attributed to N-H stretching and S-H stretching. The spectrum of Au@Cys NRs exhibited the N-H band is shifted to 1586 cm^{-1} because *L*-cysteine is modified on Au NRs and the S-H band is dispersed which converted to a strongly Au-S bond.

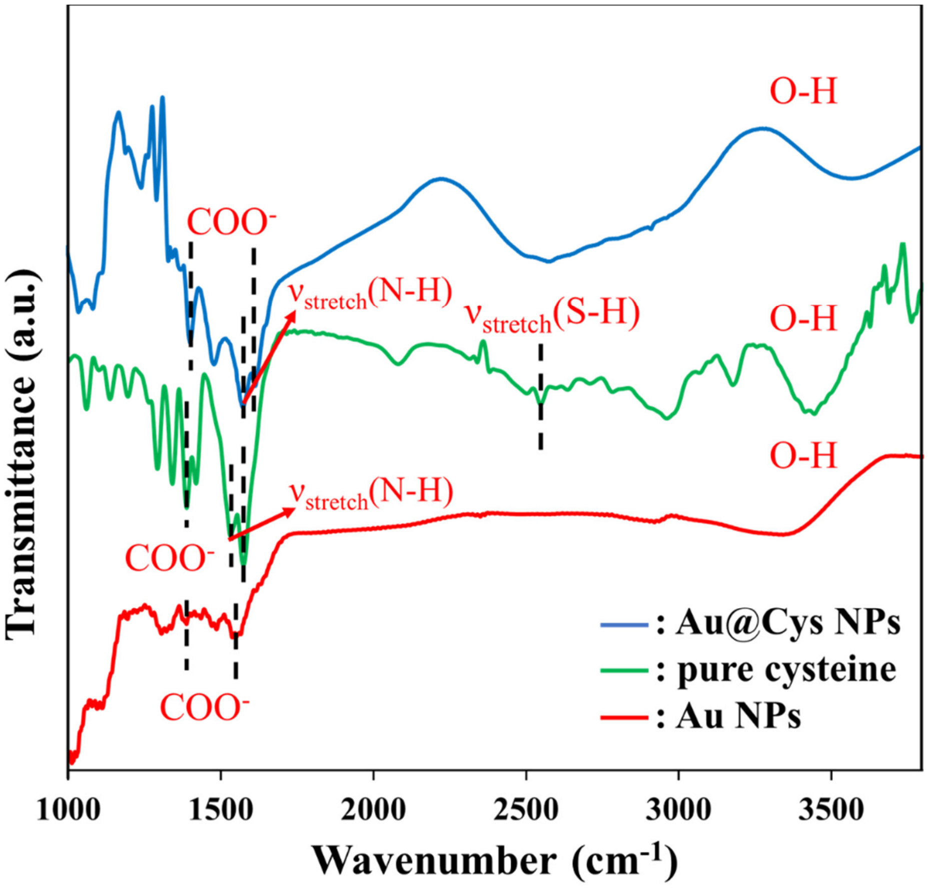

Figure S3. FT-IR spectra of pure *L*-cysteine (green line), as-prepared Au NPs (red line) and Au@Cys NPs. The peaks at 1388 cm^{-1} and 1561 cm^{-1} are assigned to asymmetric and symmetric stretching of COO$^-$ and is slightly shifted in the Au@Cys NPs condition. Then, the vibration curve of N-H band in Au@Cys NPs is shifted to 1578 cm^{-1} because of the *L*-cysteine conjugation onto Au NRs. Also, the S-H bond in the *L*-cysteine is dispersed which converted to a strongly Au-S bond.

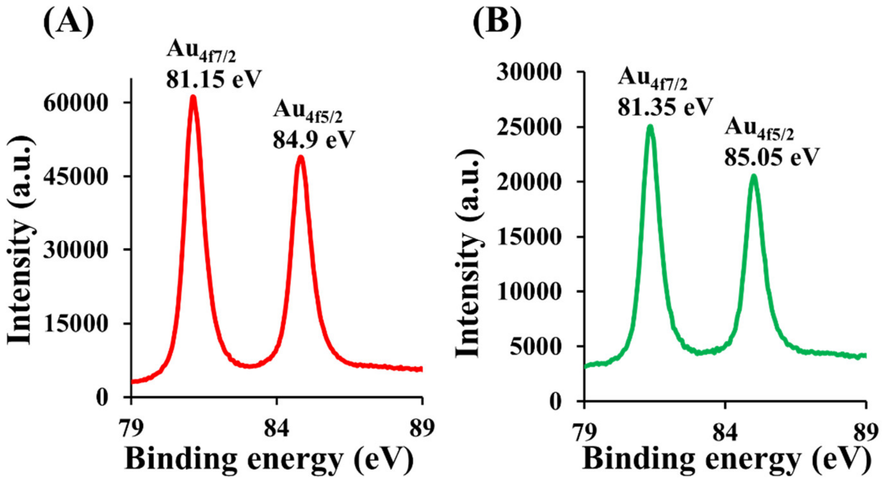

Figure S4. The XPS spectrum of Au 4f core-level of **(A)** Au NRs and **(B)** Au@Cys NRs.

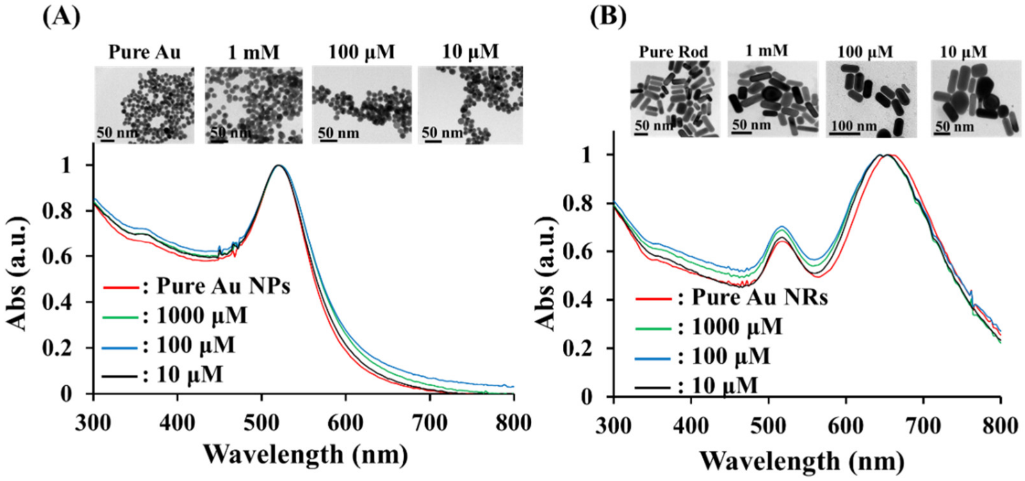

Figure S5. The UV-Vis absorbance spectrum and TEM images (insert) with four Cys concentrations (0 µM (pure Au), 10 µM, 100 µM and 1000 µM) of **(A)** Au@Cys NPs and **(B)** Au@Cys NRs after 520 nm and 650 nm laser irradiation within 4 ms.
